# People interact closer when a face mask is worn but risk compensation is at best partial

**DOI:** 10.1093/eurpub/ckad161

**Published:** 2023-09-17

**Authors:** Martin Aranguren, Alice Cartaud, Ibrahima Cissé, Yann Coello

**Affiliations:** Centre de Recherche sur les Inégalités Sociales (CRIS), CNRS and Sciences Po, Paris, France; Centre de Recherche sur les Inégalités Sociales (CRIS), CNRS and Sciences Po, Paris, France; Centre de Recherche sur les Inégalités Sociales (CRIS), CNRS and Sciences Po, Paris, France; SCALab, CNRS and Université de Lille, Lille, France

## Abstract

**Background:**

Wearing a face mask and keeping a minimal distance from others are common nonpharmaceutical interventions that governments may mandate or recommend to contain the spread of infectious diseases. The article addresses the following questions: (i) Do people interact closer when the face mask is worn? (ii) Do people interact closer because they believe that the mask reduces the risk of contagion? (iii) If the mask induces people to interact closer, does the increase in risk entailed by shorter distances entirely offset the decrease in risk offered by the mask?

**Methods:**

With a view to maximizing both the external and the internal validity of the study, between 2021 and 2022 we performed a large field experiment on real-life interactions (*n* > 4500) and a controlled laboratory experiment in virtual reality.

**Results:**

Converging between the field and the lab, the results indicate that in general people interact closer when the mask is worn, and in particular when they believe that the mask reduces the risk of contagion. However, even assuming a very low filtration efficacy and an extremely large distance-reducing effect of the mask, the counteracting effect of shorter interpersonal distances is never strong enough to entirely offset the mask’s protection.

**Conclusion:**

The distance-reducing effect of the mask is real but warrants no serious objection against a face mask policy.

## Introduction

As dramatically illustrated by the coronavirus 2019 (COVID-19) pandemic, nonpharmaceutical interventions can be crucial in the effort to contain the spread of infectious diseases. During the still ongoing COVID-19 crisis, two of the most common interventions of this type have been the recommendation or mandate to wear a face mask and to keep a minimal physical distance from others. These measures are highly generic and low cost, in the sense that they can be implemented in response to a wide array of similarly transmissible infectious diseases, in the short term and with relatively modest economic effort. However, despite their clearly beneficial potential, the relationship between wearing a face mask and keeping a minimal distance from others is still an object of controversy.

All else equal, these measures are consensually acknowledged to decrease the risk of contagion when correctly implemented.^1^^,^^2^ What is less consensual is whether the ‘all else equal’ clause is empirically plausible. More specifically, the concern has been raised that using a face mask may cause the user, or the user’s interaction partner, to decrease interpersonal distance in the context of face-to-face encounters. This offsetting between two protective measures, where the adoption of one (e.g. wearing a face mask) leads to the relaxation or abandonment of another (e.g. keeping a minimal distance), has been treated under the heading of ‘risk compensation’. Since the COVID-19 outbreak, most behavioral studies on the subject have documented a risk-compensatory effect of the face mask consisting in a tendency to decrease interpersonal distance in social interaction,^3–7^ while others have argued for its absence^8^ or even for an opposite, enhancing effect.^9^

We identify three gaps in this literature. First, although the bulk of the evidence tends to confirm it, the distance-reducing effect of the mask is still in debate. Second, whenever the mask has been shown to decrease interpersonal distance, no mechanism has been shown to account for this effect. The default explanation has been to posit that a motivation to maintain ‘risk homeostasis’^10^ must account for the distance-reducing effect of the mask, but this assumption has not been empirically examined. Third, the debate on the potential for risk compensation entailed by the interaction between mask use and interpersonal distance has tended to operate dichotomously, in an all-or-nothing manner. As a result, there has been no room for acknowledging a true unintended distance-reducing effect of the mask that is nevertheless insufficient to offset or seriously compromise its intended protective effect.

Our contribution is therefore 3-fold. First, using an original two-experiment design, we retest the hypothesis of a distance-reducing effect of the face mask both with a large field experiment on real-life dyadic interactions (*n* > 4500) and with a carefully controlled laboratory experiment. Second, in both experiments, we measure subjective risk assessments by design, with a view to examining whether ‘risk homeostasis’ accounts for the distance-reducing effect of the mask. Third, we integrate our results within a model that predicts the risk of contagion by taking into account both the protective effect of the mask and any counteracting effects of interacting at shorter distances.^11^ This model is particularly relevant for policy-making, as it provides a basis for estimating the expected net benefit of a face mask mandate.

### Distinguishing behavioral risk compensation from the hypothesis of risk homeostasis

The debate on the potential of the mask to encourage behaviors that may counteract its protective effect has been obscured by the tendency to conflate the related but distinct ideas of risk *compensation* and risk *homeostasis*. Risk compensation refers to two or more behaviors that have opposite effects on the objective probability of an undesired event, where the behavior that decreases that probability induces the behavior that increases it. Assuming that wearing a face mask decreases the objective risk of COVID-19 spread but interacting at short distances increases it, we test the hypothesis of risk compensation through the following prediction:

*P1: When the interaction partner wears a face mask, ego interacts at a shorter distance.*

The risk homeostasis construct, in contrast, operates at the psychological level and has no necessary connection with the objective probability of an undesired external event such as transmitting COVID-19. The tendency to risk homeostasis is a posited psychological mechanism to account for observable individual behaviors that manifest risk compensation. The basic tenet is that the organism strives to maintain a preferred level of perceived risk. When the organism perceives a decrease in the current level of risk, it acts to reestablish the preferred level by taking more risk and vice-versa. We test the hypothesis of risk homeostasis through the following predictions:

*P2: When the interaction partner wears a face mask, ego perceives less risk of contagion.*

*P3: When ego perceives less risk because of the mask, ego interacts at a shorter distance.*

Beyond the tendency to conflate compensation and homeostasis, another source of confusion in this debate has been the assumption that risk compensation is an all-or-nothing phenomenon, where the only relevant distinction is between protective measures that induce no offsetting behavior at all and protective measures that are totally counteracted by offsetting behaviors. But risk compensation can also be regarded as a matter of degree. In this sense, Kacelnik and Kacelnik^11^ have proposed a plausible model for predicting the probability of contagion on the basis of the combined influences of the face mask and interpersonal distance. In order to form a policy recommendation, we feed the results of our study into this model, asking the following question:

*Is the counteracting effect of shorter interpersonal distances sufficiently strong to totally nullify the increased protection offered by the mask?*

## Methods

The Research Ethics Committee of Université de Paris approved the project.

### Design and procedure

#### Field experiment

*Design.* The field experiment, conducted in the streets of Paris between July and September 2021, represents a larger and more complex version of another one conducted the prior year.^3^ Although the present report focuses on the effect of face mask use, the experiment was also conceived to test those of perceived race and social status. The field experiment follows a 2 (mask vs. no mask) × 4 (observation site) × 3 (racial group of tester: *asiatique*, *blanc*, *noir*) × 2 (social status signaled by the tester’s clothing: high vs. low) between-subjects design with random selection of participants, systematic assignment of participants to experimental conditions, and equal sampling time devoted to each of the 48 unique factor combinations. The experiment was conducted in two waves, first from 5 to 16 July and then from 6 to 17 September 2021. The CNRS DPD division certified that the procedure did not involve the collection of any identifying materials.

*Field team and sampling* (Supplementary material, Procedure). Twelve experimenters performed the assays, acting alternatively in the capacity of ‘tester’, ‘observer’ or ‘interviewer’. All experimenters spent the same amount of sampling time in each role. Unfolding in working days of dry weather, sessions began at 16:00 and ended at 19:00, covering the rush hour. In the capacity of the tester, the experimenter asked directions to pedestrians following a script. As an observer, the experimenter selected the pedestrian, assisted the tester in the performance of the script and took the measurements. The interviewer intervened after the script was complete, debriefing pedestrians and inviting them to answer a short questionnaire.

To approximate a random sample, the sampling technique exploits a random process, namely the relationship between the traffic signal’s regular alternation between red and green light, on the one hand, and the time at which pedestrians arrive at the street crossing serving as observation site, on the other. As a result of this random process, a given pedestrian’s arrival time coincides with either the green or the red period, and within these, the pedestrian is the first, second,… *n*th pedestrian to arrive in chronological order. Random selection was achieved by considering one of the outcomes of this random process: the tester interacted with the first pedestrian who came to stand still in front of the traffic light after the latter had turned red. To be eligible, the pedestrian had to be of the same gender as the tester (gender was judged by the observer’s commonsensical standards), unaccompanied, not talking on the mobile, not carrying encumbering objects (e.g. a shopping cart or a rolling luggage) and able to walk without assistance and/or particular effort.

*Procedure.* The experimenter in the role of the observer carried a rolling luggage on the top of which a cylinder (a rolled plastified map) was affixed. The cylinder acted as a standard to help the tester approach pedestrians at the one-meter distance that the authorities had recommended as a preventive measure against COVID-19 (for more details, see Ref. 3). Standing at a 1-m distance, the tester spoke to the pedestrian performing a script. In essence, the tester asked for directions; if the pedestrian did not know the answer, the tester indirectly asked the pedestrian to search the directions on their phone. The interaction ended after the answer to this indirect request. After the tester initiated the interaction at the recommended 1-m distance, the pedestrian was free to increase or reduce that initial distance, but the tester did not move anymore. The observer, who stood behind the interacting dyad, watched the exchange until the end of the script and then recorded the measures on a portable tablet.

*Treatment.* In the treatment condition, the tester interacted with the pedestrian wearing a correctly placed disposable surgical facemask. In the control condition, the tester wore no coverings at all.

*Measures.* The observer took note of some characteristics of the pedestrian and of some features of the interaction with the tester. Using their own commonsensical attributions, they assigned the pedestrian to one of three age categories (18–29, 30–49, 50– years). The observer also coded whether the pedestrian was wearing a face mask, whether the pedestrian had been approached from the left (default) or the right, and the degree to which the pedestrian had helped the tester. Additionally, the observer recorded the minimal interpersonal distance at which the pedestrian had placed himself or herself with regard to the tester in the course of the interactional episode (namely, the outcome of interest in the present study). With the aid of marks made on the cylinder that the tester used to stand at an initial one-meter distance from the pedestrian, the minimal interpersonal distance was measured on the ratio scale using a fixed set of values, namely 30, 50, 70, 90, 100, 110 and 120 cm. Inter-observer reliability was assessed by comparing nearly 100 interpersonal distance measures from each team member to those independently taken on the same units by the first author, yielding in all cases Krippendorff’s alphas above 0.7. Interpersonal distance measures are missing for approximately 10% of tested pedestrians.

After the interaction was complete, the interviewer’s questionnaire asked the pedestrian to rate the probability of having caught COVID-19 from the tester, assuming that the latter was infected with the virus. Only one fourth of the pedestrians who interacted with the tester (and for whom the observer collected behavioral measures) also answered the questionnaire.

#### Laboratory experiment

*Design.* The laboratory experiment took place at the site of IR-DIVE in the city of Turcoing. Although the present report focuses on the effect of face mask use, the experiment was also conceived to test those of trait risk aversion and speech intelligibility. The laboratory experiment follows a 2 (mask vs. no mask) × 2 (character’s sex: male vs. female) × 2 (voice: low vs. normal) × 2 (background noise: low vs. high) within-subjects design.

*Participants.* The sample size (*N* = 64, of which 21 males) was determined *a priori* for multiple regression using GPower3.1 to ensure sufficient statistical power (0.80) with α = 0.05 and a medium effect size (*f*^2^ = 0.15). Participants provided computerized informed consent and received €10 for their participation.

*Procedure.* Participants completed two tasks. In the distance task, a virtual character taken from the ATHOS database^12^ appeared at a random distance from the participant (ranging between 5.5 and 6.5 m in an empty room). The character then uttered the sentence ‘Hello, excuse me’ (taken from the script of the field experiment) with either a low or a normal voice, embedded in either a low or a normal noise. Then, participants had to virtually approach the character with a controller trackpad until they reached an appropriate distance to interact with them. Half of the time, the character appeared with a face mask. Participants were told that the characters may be the carriers of a disease transmissible by air.

In the risk perception task, participants estimated the probability of catching the character’s disease at the distances they had chosen in the previous task. Participants were presented with the character at their preferred distances in two conditions, namely with and without the mask, and were asked to rate the probability of contagion at each time.

*Measures.* Recorded on Unity, the preferred interpersonal distance measure was the distance between the character and the position of the HTC Vive pro-head-mounted display. The perceived probability of contracting the disease was also directly recorded on the software. A slider appeared in the virtual environment below the question ‘To what extent are you likely to catch the character’s disease?’, with anchor labels ‘Not likely at all’ (on the left, 0%) and ‘With certainty’ (on the right: 100%).

### Statistical analyses

The data were handled using regression models estimated with Bayesian inference, using the sampler Jags^13^ implemented in the programming language R.^14^ Prior to null hypothesis testing, with a view to preventing bias and increasing power and precision^15^ the field experimental data set, which contains a portion of missing values, was subjected to a multiple imputation procedure (Supplementary material, Imputation).

For the field data, null hypothesis testing took the shape of an instrumental variable analysis. For the laboratory data, in turn, null hypothesis testing proceeded through a two-level model with repeated measures nested within subjects as the data level and subjects as the higher level (Supplementary material, Null hypothesis testing).

### Modeling the objective probability of contagion as a function of mask use and interpersonal distance

Kacelnik and Kacelnik^11^ have proposed a model for predicting the probability of contagion of COVID-19 as a function of the interaction between the face mask and interpersonal distance. We used the results of our study as inputs in this model to evaluate the risk-compensatory effect of shorter interpersonal distances when the mask is worn. Relying on an *a fortiori* argument, we have set the parameters of the model at extreme but still credible values (Supplementary material, Risk compensation model).

## Results

We report the results of null hypothesis tests by indicating the 95% central posterior interval^16^ of the relevant coefficients. Analogous to classical confidence intervals, if the central interval excludes 0, the null hypothesis can be rejected at the conventional alpha = 5%.

### Field experiment

**Figure 1:**
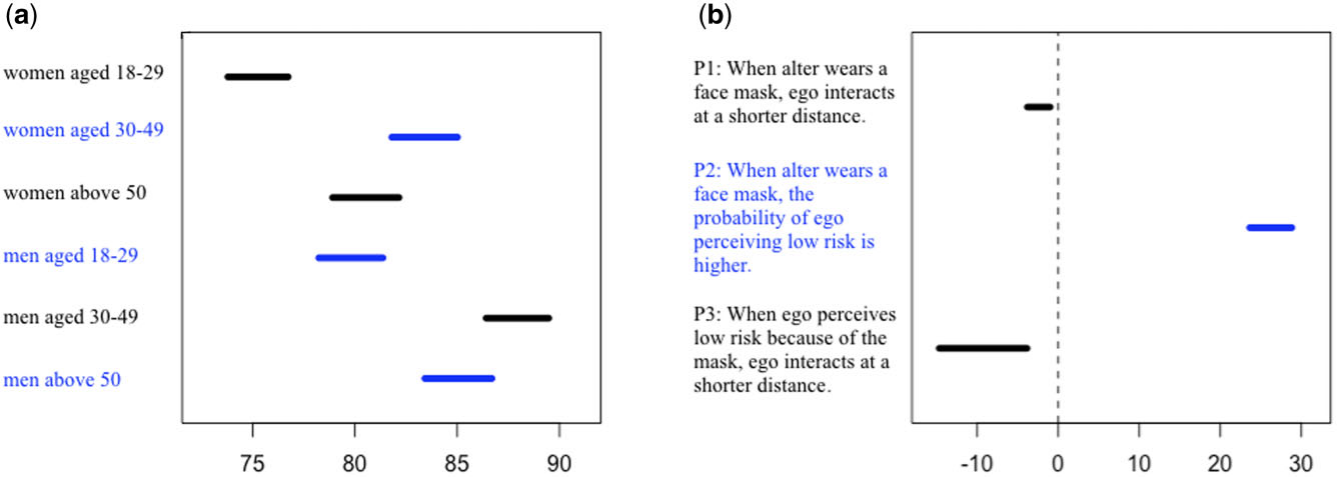
Field experiment. Segments represent 95% central posterior intervals. The labels on the left of each plot refer to estimated parameters. (a) Average interpersonal distance in cm across gender-age groups when alter does not wear a face mask. (b) Null hypothesis testing. P1 and P3 predict coefficients below 0 (indicated by the vertical dashed line). These coefficients estimate the difference in interpersonal distance, expressed in cm, between interacting with a person who is wearing vs. not wearing a face mask. P2 predicts a coefficient above 0 estimating the difference in the probability of perceiving a low risk of contagion between the mask and the no-mask condition (expressed as percentage points)

In line with P1, when the tester wears the face mask, the pedestrian decreases interpersonal distance by [−4 cm, −1 cm] (figure 1). In agreement with P2, when the tester wears the face mask, the pedestrian is [24%, 29%] more likely to perceive that the risk of catching COVID-19 is low. Supporting P3, when the pedestrian perceives a low risk of contagion because the tester wears the mask, the pedestrian shortens interpersonal distance by [−15 cm, −4 cm]. Although a portion of the values on which these results rely had to be imputed due to missingness, the conclusions remain the same when the analysis is restricted to observed values (Supplementary figure S1; figure 1).

### Laboratory experiment

**Figure 2:**
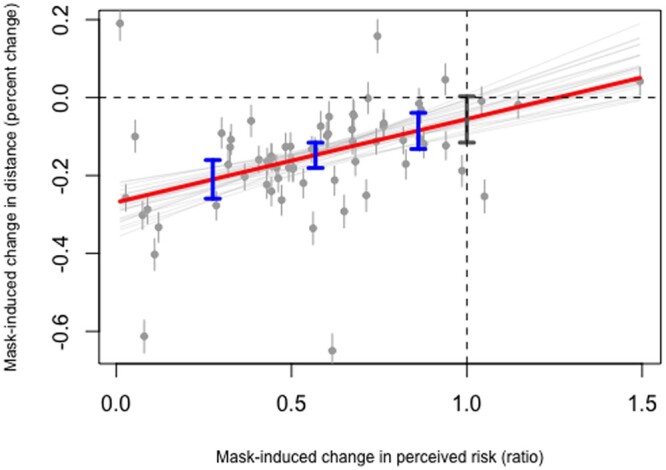
Laboratory experiment. The *x*-axis measures the within-subject change in the perceived probability of contagion between the mask condition and the no-mask condition. The *y*-axis indicates the within-subject change in interpersonal distance between the mask condition and the no-mask condition. The vertical gray segments represent the estimated effect of the mask on interpersonal distance for each of the 64 participants. The red line represents the dependency of the within-subject change in interpersonal distance induced by the mask on the within-subject change in the perceived probability of contagion induced by the mask. The intersection of the vertical and horizontal dashed lines indicates the point at which the effect of the mask is 0 both on interpersonal distance and risk assessments. The thick gray vertical segment shows that when the subject does not change their perceived probability of contagion as a result of the mask (*x* = 1), the distance-reducing effect of the mask is no longer credible at the conventional alpha = 5%. From left to right, the three blue vertical segments provide the predicted change in interpersonal distance induced by the mask at the mean minus 1 SD, the mean, and the mean plus 1 SD of mask-induced change in the perceived probability of contagion. The grey straight lines plotted around the thicker red line represent credible regression lines randomly sampled from the posterior distribution of the relevant parameters

Supporting P1, when the virtual character appears with a face mask, the participant’s preferred distance decreases on average by [−18%, −12%] (figure 2). Confirming P2, when the virtual character wears the mask, the participant’s perceived probability of contagion falls on average by [−51%, −36%]. Last, in agreement with P3, the stronger the within-subject decline in the perceived probability of contagion between the mask and the no-mask conditions, the stronger the within-subject reduction in interpersonal distance when the virtual character wears the mask.

The already reported mask-induced decrease in distance of [−18%, −12%] holds for a participant with an average change in the perceived probability of contagion from the mask to the no-mask condition. At the average minus 1 SD (i.e. for a participant whose risk perceptions were more markedly lowered by the mask), the prediction rises to [−26%, −16%], declining to [−13%, −4%] at the average plus 1 SD. When the participant’s risk assessments are unaffected by the mask, the predicted mask-induced change in distance is no longer credibly different from 0 (figure 2).

### Assessment of risk compensation

**Figure 3:**
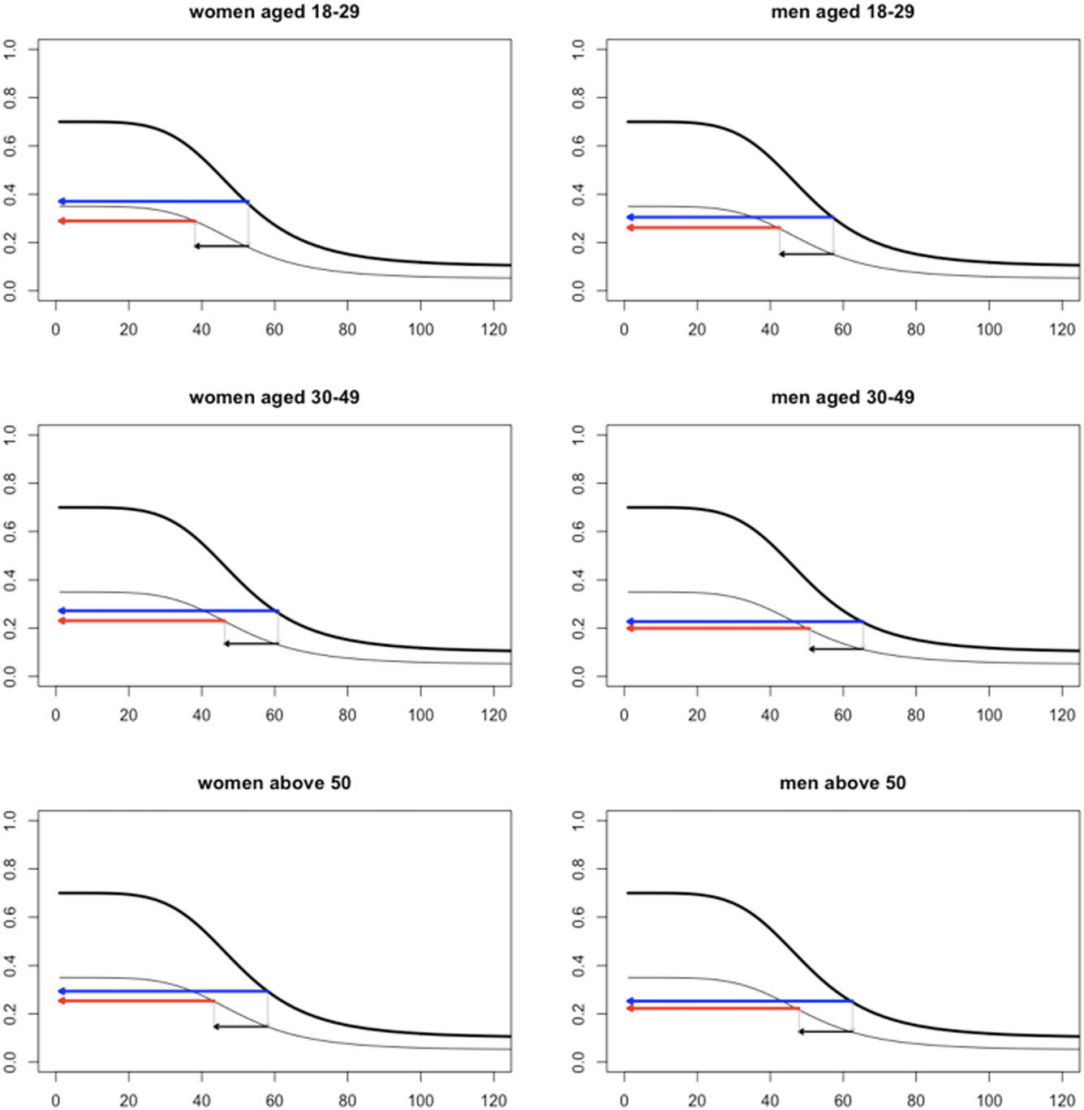
Application of Kacelnik and Kacelnik’s risk compensation model using the results of the study as inputs. The *x*-axis indicates the interpersonal distance in cm, covering the range considered in the field experiment. The *y*-axis measures the probability of contagion. We have set the upper limit of the probability of contagion at 0.7 and the mask protective effectiveness at 50%. Hence, at distance = 0, the probability of contagion is 0.7 without the mask and 0.35 with it. In each graph, the sigmoid-shaped curves (inverted S) describe how the probability of contagion declines as interpersonal distance increases. The thick sigmoid curve represents the function in the absence of the mask; the thin sigmoid curve provides the function when the mask is worn. The horizontal black arrow is constant across plots and represents the decrease in distance (in cm) elicited by the mask, as estimated from our analysis. The horizontal blue arrow indicates the risk of contagion without the mask. The horizontal red arrow indicates the risk of contagion with the mask, after taking into account the counteracting effect of the predicted decrease in distance. Full risk compensation occurs when both arrows overlap. If the red arrow remains below, that is an indication that the compensatory effect of decreased distance is only partial

Using an *a fortiori* argument, we have set the values of Kacelnik and Kacelnik’s model to credible but extreme values, assuming that if risk compensation does not occur in these extreme circumstances, it is even more unlikely to occur in a more moderate and probable scenario. Within each gender-age group, we consider the case of a person who interacts a full standard deviation closer than the average member of the same group, and for whom the effect of the mask on distance is as extreme as the lowest credible value included in the 95% central posterior interval of the parameter estimating that effect, that is −15 cm (see figure 1, P3). Another extreme assumption that we make is that the protective effect of the mask is limited to a mere 50% decrease in the risk of contagion (figure 3).

Figure 3 shows that, even in these extreme conditions, in none of the gender-age groups is the protective effect of the mask entirely offset by the counteracting increase in risk created by shorter distances. That is, the model suggests that risk compensation is at best partial.

## Discussion

In spite of marked differences in ecology and procedure, the field and the laboratory experiments converge in their results. The face mask induces a decrease in the distance at which people interact in face-to-face encounters, supporting the risk compensation hypothesis and corroborating a trend already identified in a series of previous studies. The covering also induces the perception that the risk of disease contagion is lower. And when the mask induces this perception, it also induces a reduction in interpersonal distance, supporting the risk homeostasis hypothesis. As the laboratory experiment clearly shows, when the participant’s risk perceptions are unaffected by the mask, the predicted mask-induced change in distance is zero. The more participants believe that the mask protects them, the more they decrease interpersonal distance when the interaction partner wears a mask.

However, our study also suggests that risk compensation is at best partial. We have considered the hypothetical case of people who generally interact much closer than average and for whom the distance-reducing effect of the mask is very strong, assuming that the filtration capacity of the mask is as low as 50%. Not even in this extreme scenario does the counteracting effect of shorter distances entirely offset the protective benefits of the mask. Our policy recommendation is to discard the concern that a mask mandate may inadvertently nullify the protective effects of the cover by inducing shorter interpersonal distances.

One limitation of this study is that the parameter values that we fed into Kacelnik and Kacelnik’s^11^ model are only partly based on empirical data. The sigmoid shape of the function describing the decline in the probability of contagion as interpersonal distance increases, for instance, is a reasonable choice but clearly not one resting on an unquestionable empirical basis. The data needed to fully feed the model with observed (rather than assumed) values are unfortunately not available at the time of this writing. However, we would like to emphasize that our use of this model is not intended to estimate risk compensation as it actually occurs, but more modestly to examine whether the decrease in interpersonal distance induced by the mask is enough to nullify the protective benefits of the cover *assuming credible but extremely adverse parameter values* (i.e. *very short baseline distances, very strong distance-reducing effect of the mask, very low filtration capacity of the cover*). The added value of using this model is to provide a rational basis to assess degrees of risk compensation, moving the debate beyond the received all-or-nothing dichotomy.

The two-experiment design maximizes both the internal and external validity of our concordant results. Based on a real-life task with a large sample from the general population, the field experiment guarantees ecological validity and demographic coverage. Relying on repeated observations in controlled conditions, the laboratory experiment minimizes noise and maximizes the quality of measurement. We hope that this design will be more often adopted in the future, especially when it comes to evaluating public health interventions whose effectiveness depends on the behavior of citizens in everyday situations.

## Supplementary Material

ckad161_Supplementary_Data

## Data Availability

The data underlying this article are available in the article and in its online supplementary material.
