# Supplementary material for: People interact closer when a face mask is worn but risk compensation is at best partial
Source: Eur J Public Health. 2023 Sep 17;33(6):1177–82. doi: 10.1093/eurpub/ckad161 (PMC10710335; doi:10.1093/eurpub/ckad161)
Supplement: ckad161_Supplementary_Data [file ckad161_supplementary_data.zip › ckad161_Supplementary_Data/ejph-2023-04-om-0192-File006.pdf]

Supporting Information for
People interact closer when a face mask is worn but risk compensation is at best partial

This section is structured as follows:

Supporting information text
- Procedure
- Imputation
- Null Hypothesis Testing
- Risk compensation model

Figure S1
Explanatory Memo: Dataset S1 (field experiment)
Explanatory Memo: Dataset S2 and Dataset S3 (lab experiment)
SI References

Other supporting materials for this manuscript include the following (submitted within zip file):

Dataset S1
Dataset S2
Dataset S3

Supporting Information Text

Procedure

1. Field experiment

Selection of observation sites

The observation settings are pedestrian crossings equipped with traffic lights. The selection desiderata were: the highest possible arrival rate of pedestrians, the busiest possible traffic of motor vehicles on the road to be crossed (to ensure that pedestrians would wait until the light turns to green before crossing), and a comfortable spacial arrangement for the envisaged procedure. As in the previous experiment (MASKED), the four crossings selected as observation points are located, respectively, at place de la République, place de la bataille de Stalingrad, place de Clichy, and in the vicinity of Saint-Lazare station.

Field team

All in their twenties, the twelve experimenters were equally distributed in sex/gender and race/origin categories, so that two of them were female and Asian, other two female and Black, and so on. Experimenters worked in rotating sub-teams of three; each sub-team was assigned to a different site on the same day. Of the three experimenters in which each sub-team consisted, one was locally perceived as asiatique (Asian), another as blanc (White) and another as noir (Black). Whereas in present-day Paris men in their twenties do approach men or women of all ages if they need to ask for directions, women in that

age interval are unlikely to approach men of the same age with that intent, for fear of looking “inviting” and breaching the rules of modesty in public places that are generally believed to apply to women (Condon, Lieber, and Maillochon 2005; Gardner 1990). To keep the design symmetrical and the mimicked interaction rituals in which the experimental procedure consisted as close to reality as possible, when in the role of the “tester” male experimenters interacted only with other males and female experimenters only with other females.

Imputation: Multiple imputation procedure

Number of missing values and reasons for it. In total, 4627 pedestrians interacted with one of the twelve testers. Not all trials provided complete measures on post-treatment variables. 11% of the interactions failed to satisfy the minimal conditions to measure interpersonal distance adequately. Further, 75% of the pedestrians who interacted with a tester did not answer to the questionnaire administered by the interviewer. Failing to answer to the questionnaire could be the result of two causes: the pedestrian was never asked to take part in it before they left, or the pedestrian was invited but declined. In almost all cases, pedestrians were not invited to answer to the questionnaire simply because the pedestrian light turned green again and the pedestrian took advantage of the first opportunity to resume their temporarily halted itinerary, leaving the scene even before being approached by the interviewer.

Predictors of missingness. Part of the missingness in interpersonal distance measures can be imputed to chance. A typical occurrence in this sense was that the pedestrian moved just before the tester started to speak, but not earlier enough to prevent the tester from initiating the interaction. Beyond simple chance, the strongest predictor of differences in missingness on distance as well as risk assessments is the pedestrian's decision to search on their mobile phone the street that the tester pretended to be looking for. On the one hand, this type of answer sometimes resulted in the pedestrian asking the tester to follow them to an apparently more "protected" place on a side where the search could be carried

out in more comfortable conditions. When this occurred, both pedestrian and tester left the spatial frame within which our measurement instrument could accurately describe interpersonal distance unobtrusively, accounting for a portion of the 11% of missing values on this outcome. However, there is no reason to think that the searches that unfolded outside of the instrument's restricted spatial frame differed in their proxemic profile from those that occurred within it. On the other hand, pedestrians who searched on the mobile for the tester were more likely than others to answer to the questionnaire. This is mainly imputable to the fact that these pedestrians, unlike those who provided a more succinct answer, often missed the first opportunity to cross the road. That is, the pedestrian light turned to green while they were still in the midst of the search, and by the time the street had been found, the directions given and greetings with the tester exchanged, the light had turned red again. That is, more often than in other response categories, pedestrians who sought on their phones were available for being invited to answer to the questionnaire, and this partly explains why their participation rate was higher. It is also possible (although there is no way to ascertain this) that at least a portion of the pedestrians who searched on the phone were generally more cooperative, a trait that may have manifested not only in their willingness to help the tester but also in their willingness to subsequently help the interviewer by answering to the questionnaire. These factors appear to explain why missingness overall was 75% for the variables measured through the questionnaire (including the perceived risk of contagion), but only 57% when the pedestrian searched on the phone for the tester. Age also appears to partially account for differences in missingness regarding risk perceptions. Among those who searched on

the phone, 60% of the younger (that is, a majority) answered to the questionnaire as well, but the proportion fell to 15% for the middle-aged and to 30% for the older.

Plausibility of the "missing at random" assumption. These systematic differences in missingness lend plausibility to the "missing at random" assumption, which is a necessary prerequisite for the multiple imputation procedure that we employed. This assumption implies that there are systematic differences between observed and missing values (unlike the "missing completely at random" scenario), but that these systematic differences can be accounted for by observed values (unlike the "missing not at random" scenario). In the present case, it is clear that missingness depends on the pedestrian's answer to the request for help and on the pedestrian's age, variables on which we have observed and nearly complete values. At the same time, it is hard to imagine any unobserved variables that may have systematically led pedestrians to differ in the probability of contributing a valid measure on a post-treatment variable such as distance or perceived risk. For risk perceptions, one candidate could be the interviewer wearing vs. not wearing the mask in an haphazard manner, which could have led pedestrians to decline answering to the questionnaire when the interviewer was unmasked precisely because they perceived a greater risk of contagion. But this possibility was ruled out from the beginning by instructing interviewers to wear the mask at all times.

Imputation model. For each of the post-treatment variables of interest, namely distance and perceived risk, missing values were replaced by imputed values through a regression

model estimated with Bayesian inference using the sampler Jags (ref) and R (ref). In each model the post-treatment variable was treated as the outcome and the vector of predictors included all the pre-treatment variables measured in the experiment. Each model provided a posterior probability distribution of credible predicted values for each experimental unit, that is for each pedestrian. When the prediction applied to a pedestrian for which the outcome had been observed, the prediction supplied by the model did not affect the observation. In contrast, when the prediction concerned a pedestrian for which the outcome value was missing, the posterior distribution of the predicted value was used as a "population" from which five values were randomly drawn. For each of the post-treatment variables of interest, the result is a set of five complete data vectors. Across these vectors, the values do not differ for pedestrians who did provide observations (because the observed values where unaffected by the imputation procedure), but they vary to some extent for pedestrians with missing values, because the corresponding values were randomly drawn from a population of slightly different credible values. Using a classical regression model (that is, assuming a normal distribution of the residuals), the distance measures were used as originally measured. The risk assessments were dichotomized and fed into a logistic regression. Risk assessments were originally measured on the probability scale using 10% intervals. The mode of the resulting measures is 0, with markedly lower but stable frequency between 10% and 40%, a slight spike at 50%, and declining frequency from there on. We decided to dichotomize this measure with a median split.

The 11% of missing interpersonal distance values were imputed first and the complete distance measures (that is, including both observed and imputed values) were then used as a further predictor within the model that was subsequently estimated for imputing the probability of perceiving low risk. Since predictor interpersonal distance included both observed and imputed values, the model for imputing perception of low risk was performed five times, each time with one of the five different versions of predictor interpersonal distance. The predicted probabilities used for replacing missing with imputed values were sampled from the stacked posterior distributions provided by each run of the model. The continuous predicted probabilities supplied by the logistic regression were rounded to 0 or 1. The end result of the imputation procedure for risk assessments is an indicator variable (in five versions) were value 1 indexes low perceived probability of contagion.

Null hypothesis testing

For both the field and the lab data, null hypothesis tests proceeded through regression models estimated with Bayesian inference using Jags and R. The field data were subjected to an instrumental variable analysis, whereas the lab data were modeled with a two-level regression.

1. Field experiment

Instrumental variable analysis: assumptions

Our application of instrumental variable analysis treats the mask manipulation as the *instrument*, the probability of perceiving low risk of contagion as the *treatment*, and interpersonal distance as the *outcome*. This type of analysis relies on the assumptions of 1) ignorability of the instrument, 2) nonzero association between instrument and treatment, 3) monotonicity, and 4) the exclusion restriction (Gelman & Hill, 2007). Our design and data make the four assumptions plausible.

1) Ignorability is directly satisfied by the randomization of the instrument, that is the mask manipulation.

2) As for the association between instrument and treatment, 71% of pedestrians in the mask condition perceived low risk but only 45% in the no-mask condition did so. Since we are interested in the mediating role of perceptions of low risk in the distance-reducing effect of the mask, we focus on the 26% of the pedestrians who were induced by the

mask to perceive low risk, but who would not have perceived low risk had the covering not been present. Let us call this group of pedestrians “induced low risk perceivers”. These are the only pedestrians for whom we can conceptualize a counterfactual with regard to perceived low risk of contagion.

3) The plausibility of the monotonicity assumption can be made apparent by delineating two further groups of pedestrians aside from “induced low risk perceivers”, namely “always low risk perceivers” (who perceived low risk even in the no-mask condition, i.e. when objective risk is higher) and “never low risk perceivers” (who failed to perceived low risk in spite of being in the mask condition, i.e. when objective risk is lower). Assuming monotonicity implies that i) there are no “always low risk perceivers” who would have failed to perceive low risk had they been in the mask condition, and that ii) there are no “never low risk perceivers” who would have perceived low risk had they been in the no-mask condition. There is no reason to suspect that monotonicity is violated.

4) The exclusion restriction makes the further assumption that the effect of the mask on interpersonal distance is 0 for pedestrians in the “always low risk perceiver” group and in the “never low risk perceiver” group. That is, only “induced low risk perceivers” are assumed to change their interpersonal distance because the interaction partner wears a face mask. In general, studies based on a between-subjects design such as our field experiment cannot advance data to justify this assumption and must proceed by *fiat*. But our two-experiment design, by supplementing the between-subjects field experiment with a within-subjects laboratory experiment, provides a suitable empirical basis for assessing

the plausibility of the exclusion restriction assumption. As shown in the Results section, the laboratory experiment confirms that for participants whose risk perceptions are unaffected by the mask (that is, for "never/always low risk perceivers"), the predicted change in interpersonal distance when the mask is worn is indeed 0 as assumed by the exclusion restriction.

Instrumental variable analysis: estimation

To estimate the effect of the mask on interpersonal distance among "induced low risk perceivers" we use the Wald ratio (Gelman & Hill, 2007). Let *i* denote a unique pedestrian, *y* interpersonal distance, *t* perceived low probability of contagion, and *z* the mask manipulation.

For i=1, 2, 3, …, n,

$y_i \sim \text{normal}(y.hat_i, \sigma_y^2)$, and

$y.hat_i = \beta_0 + \beta_1 * z_i + X_i\alpha$,

where $X_i\alpha$ stands for a vector of control predictors, namely the pedestrian's age (three nominal levels: 18-29/30-49/50-), the pedestrian's gender (man/woman), the observation site (four levels), the tester's perceived race (*asiatique*/*blanc*/*noir*) and the tester's perceived status (low/high). Similarly,

for i=1, 2, 3, …, n,

$t_i \sim \text{normal}(t.hat_i, \sigma_t^2)$, and

$t.hat_i = \delta_0 + \delta_1 * z_i + X_i\gamma$,

where $X_i\gamma$ stands for the same vector of control predictors as before.

Addressing Prediction 1, β_1 estimates the Average Treatment Effect (ATE) of the mask on interpersonal distance. Testing Prediction 2, δ_1 estimates the ATE of the mask on the probability of perceiving low risk of contagion. Assessing Prediction 3, the Wald ratio β_1/δ_1 estimates the local ATE of the mask for "induced low risk perceivers" under the exclusion restriction assumption.

It may be recalled that a portion of interpersonal distance values, as well as a portion of perceived low risk values, had to be imputed due to missingness in the original data set (see SM-Imputation). The end result of this imputation process is a data set with complete measures where each of these post-treatment variables appears in five versions. The instrumental variable analysis was performed by running 25 models, namely the product of 5 (observed + imputed interpersonal distance values) x 5 (observed + imputed perceived low risk values) possible combinations of these post-treatment variables. The parameters of interest, namely β_1, δ_1 and β_1/δ_1, received in each run a posterior distribution of 6000 credible values; these were stacked run after run in such a manner that the reported results provide the aggregate posterior distribution of 6000 β_1, δ_1 and β_1/δ_1 credible values per model run multiplied by 25 runs, that is the posterior distribution of 150,000 possible parameter values.

2. Laboratory experiment

Two-level model: estimation

Each of the 64 participants in this within-subjects experiment performed 16 tests in the intepersonnal distance task. Let *i* denote unique test-participant combinations, *y* the interpersonal distance outcome, *z* the mask manipulation, *j* unique participants, and *t* the by-participant average change in perceived risk between the mask and the no-mask condition (expressed as a ratio, where 1 indicates no change). For i=1, 2, 3, …, n=64 participants *16 tests=1024,

$y_i \sim \text{normal}(y.hat_i, \sigma_y^2)$, and

$y.hat_i = \beta_{0j} + \beta_{1j} * z_i + X_i\alpha$, where $X_i\alpha$ stands for a vector of test-level control predictors, namely the gender of the virtual character, the baseline virtual distance and the amplitude-noise acoustic condition.

In turn, for j=1, 2, 3, …, k=64,

$\beta_{1j} \sim \text{Student t}(\beta_1.hat_j, \sigma_{\beta 1}^2, \nu_{\beta 1})$,

and $\beta_1.hat_j = \delta_0 + \delta_1 * t_j$. The Student t distribution instead of the normal-gaussian was preferred to prevent outliers from distorting estimates. For the same participants *j*,

$t_j \sim \text{normal}(t.hat_j, \sigma_t^2)$, and

$t.hat_j = \gamma_0 + \gamma_1 * z_j + X_i\zeta$, where $X_i\zeta$ stands for a vector of participant-level control predictors.

Testing Prediction 1, the mean of β_{1j} estimates the ATE of the mask on interpersonal distance. Addressing Prediction 2, γ_1 estimates the ATE of the mask on by-participant changes in perceived risk of contagion. Assessing Prediction 3, δ_1 assesses the degree to which the participant-level change in perceived risk induced by the mask predicts the

participant's change in interpersonal distance when interacting with the mask-wearing virtual character.

The *y* outcome, interpersonal distance measured in cm, was transformed into its logarithm to enhance the data-level model's fit. Taking advantage of the possibility of interpreting coefficients as percentages in the resulting log-normal model (Gelman & Hill, 2007), we report the effect of the mask on distance as a predicted percentage change. These models were estimated in 30,000 runs.

Risk compensation model

Kacelnik and Kacelnik's (2022) model

The model predicts the probability that an infected person transmits the disease to a healthy person as a function of the distance at which they interact. This probability declines as distances increases, following the sigmoid function defined in the following equation:

(1)

probability of contagion at distance D = $(C_{max} + C_{min} * D_{1/2}^{-k} * D^{k}) / (1 + D_{1/2}^{-k} * D^{k})$,

where

- C_{max} is the upper limit of the probability of contagion. If C_{max} is set at 0.7, that means that when distance D is 0, the probability of contagion is 70%.
- C_{min} is the lower asymptote of the transmission probability, that is the baseline probability of contagion when the influence of interpersonal distance is null. If C_{min} is set at 0.1, that means that when the risk of contagion falls to 10%, distance D no longer increases the baseline risk in the environment.
- $D_{1/2}$ designates the distance at which the probability of transmission is halfway between C_{max} and C_{min}, and therefore the point in the curve where decline is steepest.
- Parameter *k* controls the sensitivity of contagion to distance, and therefore the steepness of the descending curve.

The net probability of transmission at a given distance *when the mask is worn* takes into account the filtration efficacy of the cover and any distance-reducing effect it may have.

(2)

probability of contagion at distance D when the mask is worn=

$\alpha * (C_{max} + C_{min} * D_{1/2}^{-k} * (\beta * D)^k) / (1+ D_{1/2}^{-k} * (\beta * D)^k)$,

where (1-α) refers to the filtration efficacy of the mask. For example, if the filtration capacity of the cover is 90%, α=10% or 0.1. Finally, β designates the change in interpersonal distance induced by the mask.

The present application

We introduce a slight modification to equation (2). To make the model isomorphous with our empirical analysis, we replace the β coefficient, which acts as a multiplier of D, with a δ difference that adds a fixed quantity to distance D, so that

(3)

probability of contagion at distance D when the mask is worn=

$\alpha * (C_{max} + C_{min} * D_{1/2}^{-k} * (\delta + D)^k) / (1+ D_{1/2}^{-k} * (\delta + D)^k)$

Recall that in analyzing the field experimental data, the ATE of the mask on interpersonal distance was estimated as a fixed difference. Given the limited range of observed

interpersonal distances evidenced in the field data, this additive interpretation of the effect of the mask turns out to offer a better fit than the alternative multiplicative interpretation (which is not the case for the lab data, where the range of virtual distances considered extends to several meters).

In order to construct an extreme but still credible scenario, we keep some of the values that Kacelnik and Kacelnik chose for illustrative purposes, namely C_{max}=0.7, C_{min}=0.1 and k=5. To force the influence of interpersonal distance on the probability of contagion to virtually disappear at the one-meter distance that the French authorities had recommended (that is, to adjust the world to the dictates of the French government), we set $D_{1/2}$ at 50 cm. One extreme assumption that we make is that the filtering capacity of the mask is limited to a mere 50%, so that α=0.5.

We make the remaining extreme assumptions on the basis of credible estimations from our study.

We set the δ difference in interpersonal distance induced by the mask to the lower bound of its 95% estimated central posterior probability interval, that is -15 cm. We then set the distance D at the mean of each gender-age group minus one σ_y as estimated from the field data (see SM-Method-Null Hypothesis Testing). In other words, we evaluate risk compensation for a person who interacts one full sigma closer than the average member of the group (implying that nearly 70% of the group interacts farther), and assuming that the distance-reducing effect of the mask is the largest of all the credible values considered in the analysis.

Supplemental Materials, Results

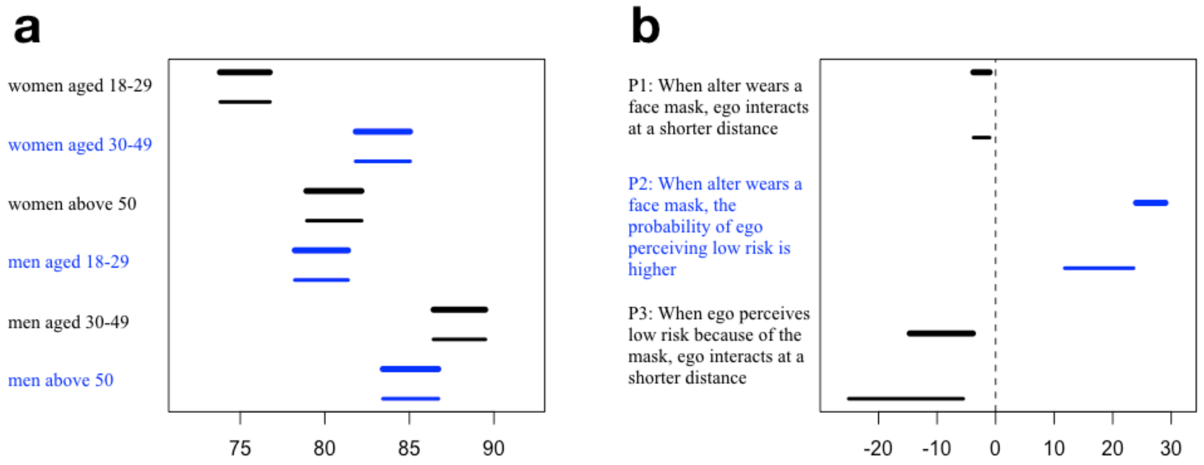

Figure SM1: Field experiment. Segments represent 95% central posterior intervals. The labels on the left of each plot refer to estimated parameters. Each was estimated on the basis of both observed and imputed values (thick segment) and on the basis of data restricted to the observed values (thin segment). **Pane a: Average interpersonal distance in cm across gender-age groups when alter does not wear a face mask. Pane b: Null hypothesis testing.** P1 and P3 predict coefficients below 0 (indicated by the vertical dashed line). These coefficients estimate the difference in interpersonal distance, expressed in cm, between interacting with a person who is wearing vs. not wearing a face mask. P2 predicts a coefficient above 0 estimating the difference in the probability of perceiving low risk of contagion between the mask and the no-mask condition (expressed as percentage points).

Dataset S1 (separate file)

Statistical analysis: explanatory memo

Field experiment

Description	Data file	Syntax file	Log file
imputation of missing interpersonal distance values	dataJulySep2021_OK.csv	1.imputationProcedure-ipd.R	dataJulySep2021_COMPLETE-IPD.csv
imputation of missing perceived low risk values	dataJulySep2021_COMPLETE-IPD.csv	2.imputationProcedure-lowRisk.R<br>> 2a.imputationProcedure-lowRisk-model.R	dataJulySep2021_COMPLETE-IPD-lowRisk.csv
instrumental variable analysis: estimation (observed and imputed data)	dataJulySep2021_COMPLETE-IPD-lowRisk.csv	3.instVarAnalysis.R<br>> 3a.instVarAnalysis-model.R	instVarAnalysis-postDist.csv
instrumental variable analysis: estimation (only observed data)	dataJulySep2021_OK.csv	4.instVarAnalysis-restricted.R<br>> 3a.instVarAnalysis-model.R	instVarAnalysis-restricted-postDist.csv
instrumental variable analysis: plotting	instVarAnalysis-postDist.csv<br>instVarAnalysis-restricted-postDist.csv	5.instVarAnalysis-plotting.R<br>6.instVarAnalysis-plotting-COMPLETEvsRESTRICTED	[R plot file]

Note: files preceded by symbol “>” are embedded within the file not preceded by this symbol.

Dataset S2 and Dataset S3 (separate files)

Statistical analysis: explanatory memo

Laboratory experiment

Description	Data file	Syntax file	Log file
two-level model: estimation	raw_t1.Rda<br>raw_t2.Rda	lab-Multilevel.R	[selected parameters posterior distribution]
two-level model: plotting	[selected parameters posterior distribution]	subjectLevelModelPlot.R	[R plot file]

SI References

Gelman, A., & Hill, J. (2007). Data analysis using regression and multilevel/hierarchical models. Cambridge University Press.

Kacelnik, O., & Kacelnik, A. (2022). Behavioral risk compensation and the efficacy of nonpharmacological interventions. *Behavioural Public Policy*, *6*(1), 1–12. https://doi.org/10.1017/bpp.2021.1
